# The Intricate Functional Networks of Pre-mRNA Alternative Splicing in Mammalian Spermatogenesis

**DOI:** 10.3390/ijms252212074

**Published:** 2024-11-10

**Authors:** Nan Jiang, Yi Li, Lisha Yin, Shuiqiao Yuan, Fengli Wang

**Affiliations:** Institute of Reproductive Health, Tongji Medical College, Huazhong University of Science and Technology, Wuhan 430030, China; m202275824@hust.edu.cn (N.J.); 15211455176@163.com (Y.L.); lisha_yin1999@163.com (L.Y.)

**Keywords:** alternative splicing, spermatogenesis, male infertility

## Abstract

Spermatogenesis is a highly coordinated process that requires the precise expression of specific subsets of genes in different types of germ cells, controlled both temporally and spatially. Among these genes, those that can exert an indispensable influence in spermatogenesis via participating in alternative splicing make up the overwhelming majority. mRNA alternative-splicing (AS) events can generate various isoforms with distinct functions from a single DNA sequence, based on specific AS codes. In addition to enhancing the finite diversity of the genome, AS can also regulate the transcription and translation of certain genes by directly binding to their cis-elements or by recruiting trans-elements that interact with consensus motifs. The testis, being one of the most complex tissue transcriptomes, undergoes unparalleled transcriptional and translational activity, supporting the dramatic and dynamic transitions that occur during spermatogenesis. Consequently, AS plays a vital role in producing an extensive array of transcripts and coordinating significant changes throughout this process. In this review, we summarize the intricate functional network of alternative splicing in spermatogenesis based on the integration of current research findings.

## 1. Introduction

Currently, infertility poses a significant public health challenge, affecting one in eight couples, with approximately half of infertility cases attributed to male factors [1,2,3]. The prevalence of infertility among men is reported to be around 12%, often resulting from secondary hypogonadism, obstructive azoospermia, or testicular dysfunction [4]. In this manuscript, we shed light on one of the major factors in testicular dysfunction—genetic anomalies. Numerous genes work collaboratively during spermatogenesis, where specific genetic disturbances lead to distinct molecular and cellular processes, yet similar phenotypes manifest as nonobstructive azoospermia [5].

Spermatogenesis, a meticulously orchestrated process that produces male gametes, generally can be divided into three phases: the maintenance and differentiation of spermatogonia, meiotic division of spermatocytes, and maturation of haploid gametes. Before releasing mature sperm into the epididymis, the cells with testes undergo significant transcriptional and translational dynamics [6]. Alternative splicing (AS) plays a crucial role in male gamete biogenesis, contributing to the sophisticated molecular activities that result in one of the most complex tissue transcriptomes. The long-held notion of “one gene, one polypeptide” has been challenged; AS significantly increases the diversity and complexity of the transcriptome and proteome derived from a finite genome [7,8]. Recent advancements in high-throughput sequencing have identified numerous transcript variants and several modes of alternative splicing. The five major modes of AS include exon skipping, intron retention, mutually exclusive exons, alternative 5′ splice sites, and alternative 3′ splice sites, which collectively explain why the number of proteins exceeds the number of protein-coding genes [9]. In the human genome, 21,144 multiexonic protein-coding genes can generate 215,170 isoforms, averaging 3.4 isoforms per gene [10]. Approximately 95% of multiexon genes undergo splicing in humans, compared to around 63% in mice [10]. Approximately 95% of multiexon genes undergo splicing in humans, compared to around 63% in mice [11,12,13]. Any alteration in the intrinsic alternative splicing of genes—such as aberrant AS events—can impact mRNA stability, translation, and localization [14].

In this review, we synthesize recent research on alternative splicing to elucidate its role in male spermatogenesis (Table 1). We explore the interplay between AS regulators and various testis-specific key factors that contribute to the production of mature haploid gametes, as well as how dysregulation of AS can lead to male infertility and other human diseases.

## 2. The Mechanism of Alternative Splicing and Related Functional Pathways

### 2.1. How Alternative Splicing Works During the Molecular Biological Process

**Figure 1:**
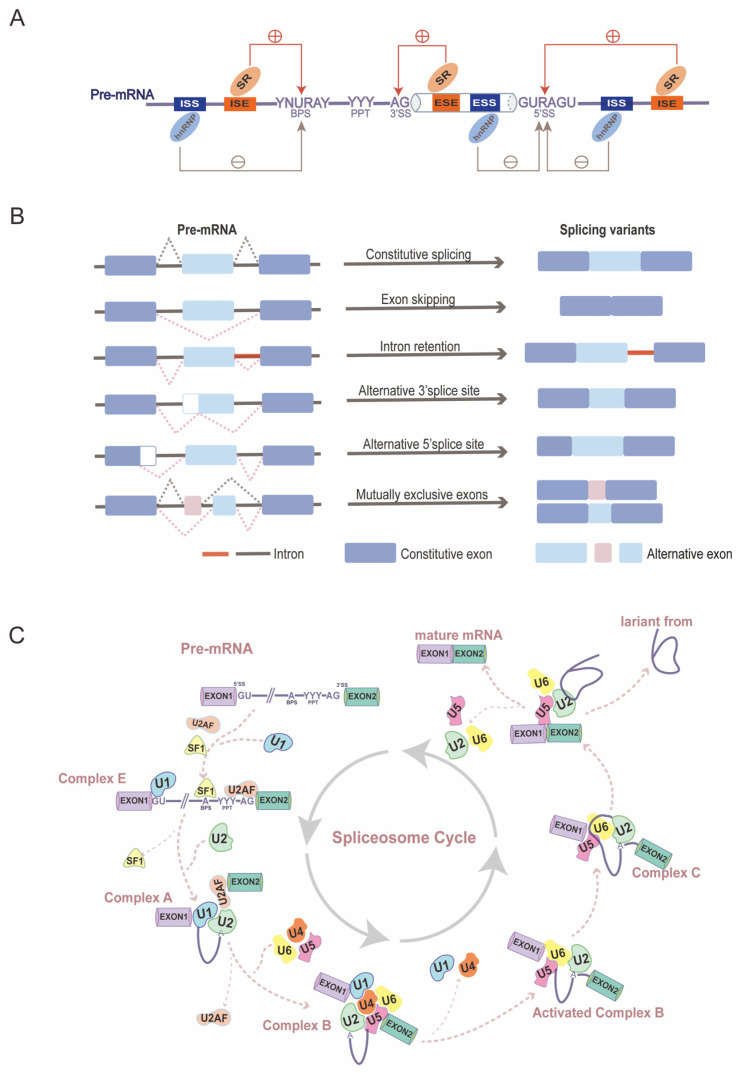
The mechanism of alternative splicing: (**A**) *cis*-acting splicing-regulatory elements (SREs) and *trans*-acting splicing factors. Abbreviation, exonic splicing enhancers (ESEs), silencers (ESSs), intronic splicing enhancers (ISEs), intronic splicing silencers (ISSs), serine/arginine-rich (SR) proteins, and heterogeneous nuclear ribonucleoproteins (hnRNPs) [55]. (**B**) Consecutive splicing and five major modes of alternative splicing. (**C**) Spliceosome cycle [14].

Once RNA polymerase II initiates binding to the DNA template, RNA synthesis begins, resulting in the transcription of genes into precursor messenger RNAs (pre-mRNAs) that contain intron-interrupted coding sequences [48]. For pre-mRNAs to mature into functional mRNAs, introns must be excised, and exons must be joined together, a process catalyzed by specific nucleotide sequences and spliceosomes [49]. The spliceosome, which is central to the mechanism of alternative splicing, is a macromolecular ribonucleoprotein complex composed of five small nuclear ribonucleoproteins (U1, U2, U4, U5, and U6) along with various auxiliary proteins [50]. The complex process of alternative splicing can be visualized as a cycle of spliceosome’ s assembly and disassembly, which occurs repeatedly after each time an intron is excised from a pre-mRNA (Figure 1C). The main steps in the spliceosome cycle can be summarized broadly: in the first step, formation of complex E: U1 snRNP binds to the 5′ splice site, while splicing factor 1 (SF1) and U2 auxiliary factor (U2AF) attach to the branch point site (BPS) and polypyrimidine tract (PPT), respectively; secondly, U2 snRNPs (small nuclear ribonucleoproteins) then replace SF1 via base pairing, proceeding to complex A; thirdly, after the release of U2AF, the U4/U6/U5 tri-snRNP is recruited, forming a pre-catalytic spliceosome, known as complex B; next, U1 and U4 snRNP are released, activating the complex B; consequently, after two rounds of transesterification reactions, introns are folded into a lariat, two adjacent exons are joined, and snRNPs enter next cycle [14,51,52,53,54]. In addition to the splicing process itself, five major modes of alternative splicing deserve detailed mention: exon skipping, intron retention, mutually exclusive exons, alternative 5′ splice sites, and alternative 3′ splice sites (Figure 1B) [54].

### 2.2. Splicing Regulators in Alternative Splicing

Alternative splicing is regulated by the interplay between *cis*-acting splicing-regulatory elements (SREs) and *trans*-acting splicing factors [56]. *cis*-acting splicing-regulatory elements are consensus nucleotide sequences found in the exons and introns of pre-mRNA, determining the removal or retention of specific exons. These elements include binding sites for trans-acting factors and guide the assembly of spliceosomal complexes. *cis*-acting splicing-regulatory elements comprise exonic splicing enhancers (ESEs) and silencers (ESSs), as well as intronic splicing enhancers (ISEs) and silencers (ISSs) [55,57]. *Trans*-acting splicing factors comprise a vast group of RNA-binding proteins (RBPs), primarily including two families involved in the splicing process: serine/arginine-rich (SR) proteins and heterogeneous nuclear ribonucleoproteins (hnRNPs). SR proteins are recruited by exonic splicing enhancers (ESEs) and intronic splicing enhancers (ISEs) to promote splicing, while hnRNPs bind to ISEs and intronic splicing silencers (ISSs) to inhibit splicing [58,59]. Typically, SR and hnRNP proteins exert opposing effects on the regulation of cellular RNA splicing (Figure 1A). Notably, many members of these two families have been identified as playing critical roles in spermatogenesis through mechanisms dependent on alternative splicing, which will be discussed in greater detail later [54,56,60,61].

### 2.3. Crosstalk Between Other RNA Alternative Splicing and Metabolism Regulators in Spermatogenesis

The fidelity of alternative splicing is under stringent scrutiny as it ensures the proper proteins production, which is indispensable for cell growth and biological development [62,63]. However, owing to the transcripts’ complexity and diversity, mis-spliced isoforms are frequently inevitable and obliged to be eliminated from the mRNA pool that is ready to be translated into proteins. Otherwise, the translation of aberrantly spliced transcripts may give rise to toxic truncated proteins, which pose a threat to cellular and molecular homeostasis [16,64]. Nonsense-mediated mRNA decay (NMD) plays a key role in identifying and eliminating faulty transcripts containing premature termination codons (PTCs) or unusually long 3′ untranslated regions (3′UTRs). SRSF1, an essential splicing factor, promotes NMD when located downstream of a premature termination codon [65]. UPF2, a key factor of NMD pathway, is essential for clearing mRNAs with PTCs and long 3′UTRs and is necessary for Sertoli cell development and spermatogenesis in male mice [15,66]. Bao et al. proved that ablation of UPF2 in Sertoli cells causes a Leydig-cell-only phenotype by decreasing nine core *trans* elements, including Wt1 and Dmrt1, contributing to the differentiation of Sertoli cells [15]. Another key factor in the NMD pathway is a pair of homologs—UPF3A and UPF3B—the latter of which co-localizes with the chromatoid body (CB) in round spermatids. Fanourgakis et al. illustrated that the depletion of TDRD6–CB components disrupts the recruitment of UPF1 to mRNAs carrying long 3′UTR and contributes to higher mRNA stability via decreased degradation in post-meiosis haploid [16]. The correlation between key components of NMD and CB suggests that surveillance of alternative splicing may also be affected by CBs, the germ granules in post-meiosis haploids, loaded with RNA and RNA-binding proteins [67,68]. Furthermore, several clinical cases of infertile men, diagnosed as oligo-astheno-teratozoospermia (OAT), have been reported to carry TDRD6 variants, and their symptoms are consistent with phenotypes in male mice [17,69,70]. In these cases, six men carrying TDRD6 variants, together with their wives, were treated with intracytoplasmic sperm injection (ICSI), but all failed due to low fertilization rates and poor quality of the embryos. Binbin Wang’s group mentioned that they would try to use artificial oocyte activation (AOA) to improve fertilization rates in the subsequent ICSI cycles, which deserves our attention [70].

N6-methyladenosine (m6A), the most common modification in mRNA, is involved in all stages of RNA metabolism, including the transcription, maturation, transportation, translation, degradation, and stability of mRNA, in mammals [71,72]. Recent studies have illuminated the interplay between m6A modifications and alternative splicing, particularly in the context of tumorigenesis [73]. The m6A modification can mediate alternative-splicing functional patterns by recruiting various RBPs to their target genes or directly affecting the binding dynamics between RNA and RBPs. In turn, alternative splicing can regulate the m6A’s deposition on specific mRNA by m6A methyltransferases (m6A writer) and recognition by the m6A reader. Key m6A regulators have been shown to significantly impact spermatogenesis through their interaction with alternative splicing. METTL3, the earliest identified m6A methyltransferases, can form a stable heterodimer with another m6A methyltransferases-METTL14, facilitating m6A deposition on specific RNAs in the nucleus [74]. Previous studies have demonstrated that genes with m6A deposition show a high level of alternative exon inclusion [75,76]. The depletion of METTL3 caused decreased exon inclusion level of aberrant AS events of several key genes *(Dazl*, *Sohlh1*, *Cdk11b*, and *Nasp*) in spermatogenesis, which then contributed to a defected initiation of spermatogonia differentiation [18].

Moreover, METTL16, the known methyltransferase for U6 spliceosome small nuclear RNA (snRNA), can install the N6-methylation to A43 of U6 snRNA, located in a fundamental domain of U6 spliceosome, which base pairs 5′ splice sites in pre-mRNA during splicing process [77]. During mice spermatogenesis, METTL16 interacts with SF3B1 and SF3B1 to modulate alternative splicing of meiosis-related genes such as *Stag3*, *Stra8*, and *Ddb2*, while the absence of METTL16 leads to impaired spermatogonia differentiation and compromised meiosis initiation [19]. ALKBH5, the m6A demethylase (m6A eraser), is necessary to generate longer 3′UTR mRNAs with appropriate m6A modifications in male spermatogenesis. In the post-meiosis phase, the longer the 3′UTR transcripts are modified with m6A, the more easily they will be degraded. Indeed, under physiological conditions, the genome-wide shortening transcripts are required for high translation efficiency when transcription activity is shut down during late spermiogenesis [78]. Inactivation of ALKBH5 fails to remove m6A from long 3′UTR mRNAs, contributing to enhanced splicing (up-regulated ESI and down-regulated ISR) and production of massive mis-spliced shorter 3′UTR mRNAs with m6A, which tend to be degraded soon [20]. This process aligns with the previously discussed NMD pathway, where long 3′UTR mRNAs are targeted for degradation [66]. In summary, the regulation of alternative splicing through mechanisms like NMD and m6A modification is essential for maintaining proper cellular function and development, particularly in spermatogenesis.

## 3. Alternative Splicing in the Self-Renewal and Differentiation of Spermatogonia

**Figure 2:**
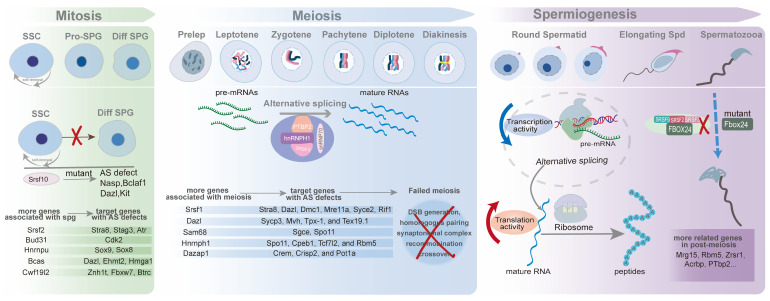
Alternative splicing in each stage of spermatogenesis. To begin with, germ cells undergo mitosis to give rise to a certain amount of spermatogonia, including undifferentiated and differentiated spermatogonia. Then, differentiated spermatogonia develop into pre-leptotene spermatocytes, entering meiosis, one of most intricate process in biological and molecular activities. After premise and multi-steps of two-round cell divisions, the haploid must go through chromatin condensation, elongation, flagella and acrosome formation, and cytoplasmic elimination. Besides depicting major processes of spermatogenesis, we further delineate one of its representative genes’ schematic diagram in each substage, for example, *Srsf10* in mitosis, *Hnrnph1* in meiosis, and *Fbox24* in spermiogenesis [33,47,81]. Meanwhile, we also list some critical genes and their target genes in its corresponding phase.

In the gonads of male embryonic mice, primordial germ cells transform into T1-prospermatogonia (ProSG) by E12.5, and then T1-ProSG enters a state of arrest at the G0/G1 phase of the mitotic cell cycle by E15. After birth, T1-ProSG migrates from the center of the lumen towards the peripheral basement membrane, regaining its proliferative capacity to develop into T2-ProSG within three days [79,80]. Some T2-ProSGs differentiate into spermatogonial stem cells (SSCs), while others directly become A2 spermatogonia-differentiating spermatogonia, which subsequently undergo meiosis, initiating the first wave of spermatogenesis [81]. Bypassing SSCs in the development process is the most remarkable characteristic of the first wave of spermatogenesis different from constant waves in male mice. SSCs are a specialized cell type within the male germline, possessing the dual potential for progressive differentiation and continuous self-renewal. This population, defined as the undifferentiated spermatogonia—the most primitive cells in the mammalian testicular germ cell lineage—includes A_single_ (A_s_), A_paried_ (A_pr_), and A_aligned_ (A_al_) spermatogonia, named based on the number of cells in their clones and interconnected by intercellular cytoplasmic bridges [82]. The maintenance of this population, defined as the stem cell pool, is a prerequisite for sustaining ongoing spermatogenesis and male fertility. SSCs partially proliferate into differentiating spermatogonia (A1-4, Intermediate, and B spermatogonia) while retaining a portion of their population [79]. Further, type B spermatogonia differentiate into spermatocytes, which in turn go through meiosis to generate genetically diverse gametes (Figure 2).

SRSF10, a member of the SR protein family, is highly expressed in the brain and testis and participates in many essential cellular and molecular processes via regulating accurate alternative splicing. Liu et al. demonstrated that SRSF10 is vital for maintaining alternative splicing (AS) homeostasis during spermatogenesis [21]. Deletion of SRSF10 impedes the differentiation of progenitor spermatogonia, which are meant to develop into differentiating spermatogonia. This disruption severely affects both transcription and post-transcriptional alternative splicing of RNA in progenitors, ultimately impairing meiosis initiation in male mice. The knockout of SRSF10 caused multiple differentially spliced events (DSEs). Moreover, genes involved in DSEs play crucial roles in RNA metabolism, meiosis, basic biological process, translation regulation, such as *Dazl* (increased exon inclusion) [83], *Sycp1* (increased exon skipping) [84], *Kit* (A5SS) [85], *Exo1* (A5SS) [86], *Ret* (alternative last exon) [87], and *Cdc7* (increased exon inclusion) [88].

Additionally, SRSF1 has been extensively studied in relation to alternative splicing in contexts such as tumorigenesis, heart development, and immune organ development, highlighting its broad significance in various biological processes [22,89,90]. In the testis, SRSF1 plays crucial roles in the homing of precursor spermatogonial stem cells (SSCs) and the survival of spermatogonia by directly binding and regulating the expression levels of TIAR through an alternative splicing pathway [91]. Additionally, SRSF1 interacts with other splicing factors such as SART1, RBM15, and SRSF10, mediating the regulation of genes essential for spermatogonial development. SRSF2, another member of the SR protein family, directly affects the abundance of mRNAs for key genes like *Stra8*, *Stag3*, and *Atr* while increasing the exon skipping ratio of their pre-mRNAs during alternative splicing. SRSF2 knockout mice show arrested spermatogonia differentiation, leading to failed meiosis initiation [23].

BUD31, key component of complex B of mammalian spliceosome, is essential for spliceosome cycle. Deficiency of BUD31 highlights the importance of spliceosomal components in maintaining the SSC reservoir and initiating meiosis in an alternative splicing-dependent manner [24]. Specifically, BUD31 depletion results in the retention of the first intron of *Cdk2* pre-mRNA and decreased expression of CDK2 at post-transcriptional level, indicating that precise regulation of this gene is vital for the transition from gonocytes to spermatogonia [25]. Previous study has demonstrated that intron retention leads to the down-regulated gene expression post-transcriptionally [92]. CWF19-like protein 2 (CWF19L2), functioning particularly and necessarily in the final stage of spliceosome cycle to form a complex with intron lariat, is evolutionarily conserved from yeast to mammalians [93,94,95]. Wang et al. illustrated that absence of CWF19L2 resulted in massive apoptosis in differentiating spermatogonia with no meiocytes observed, while undifferentiated spermatogonia remained unaffected. Furthermore, CWF19L2 can regulate alternative splicing by directly binding to genes involved in spermatogenesis (such as *Znh1t*, *Btrc*, and *Fbxw7*) and RNA splicing (including *Rbfox1*, *Celf1*, and *Rbm10*). Notably, approximately 85% of the genes involved in differentially alternative spliced events were not direct targets of CWF19L2, suggesting that CWF19L2 may modulate the alternative splicing of other splicing factors, like RBFOX1, leading to widespread changes in alternative splicing levels [26].

UHRF1, known as ICBP90 in human and NP95 in mice, is a key player in epigenetic mechanism network involved in oocytes maturation, early embryonic development, and spermatogenesis [96,97]. Recent research has revealed a novel role for UHRF1 in RNA metabolism, demonstrating that it can directly bind to U1, U2, and U4 snRNPs and interact with a wide array of ribonucleoproteins (RNPs) to modulate the alternative splicing of genes related to spermatogonial development. The depletion of UHRF1 led to impaired spermatogonia differentiation and SSCs homeostasis, contributing to the development of Sertoli-cell-only syndrome [27]. HnRNPU, the largest member of hnRNP protein family, significantly influences pre-pubertal Sertoli cell proliferation, development, and postnatal maturation, as well as the migration and differentiation of pro-spermatogonia [28,29]. The depletion of hnRNPU in Sertoli cells led to testis atrophy and degradation of seminiferous tubules, resulting in the presence of Sertoli-like cells and germ-cell-like cells. Mechanistically, hnRNPU binds to *Sox8* and *Sox9*’s promoter region, enhancing their expression. For another, hnRNPU interacts with SOX9 and WT1, thereby dominating Sertoli cell’s evolution and male fertility in mice [28]. The same group later confirmed that the absence of hnRNPU in pro-spermatogonia caused an arrest at the transition from T1-ProSG to T2-ProSG, affecting the migration of pro-spermatogonia. Specifically, hnRNPU interacts with RNA-binding proteins and binds to pre-mRNA to mediate its AS events during early spermatogonial development [29]. The loss of PTBP1 in spermatogonia resulted in approximately 20% of the tubules showing disorganization, while NANO3 knockout exhibited impaired pro-spermatogonia differentiation. More importantly, double knockout of PTBP1 and NANO3 resulted in a complete absence of germ cells, including spermatogonia (PLZF+), spermatocytes (SYCP3+), and spermatids. Notably, PTBP1 can bind to the mRNA of *Nano3*, modulating alternative splicing and the transcriptome in spermatogonia through multiple pathways [30,31].

## 4. Alternative Splicing in Meiotic Division of Meiocytes

Meiosis is one of the most intricate and ingeniously regulated processes in eukaryotic biological activities, during which genetic material undergoes a single replication followed by two successive cell divisions [98]. The prophase of meiocytes can be divided into five substages based on the molecular behavior and status of chromatin. Initially, chromatin is remodeled and condensed into a linear array of loops emanating from well-coordinated structural chromosome axes. Subsequently, numerous programmed double-strand breaks occur at hotspots within the chromatin loops during leptotene, promoting recombination and homologous synapsis in later prophase [98,99,100]. Following leptotene, zygotene is marked by critical events such as homologous pairing and the formation of chromosome synapsis complexes, driven by complex molecular and genetic forces. With telomeres attached to the inner nuclear envelope and clustered into a bouquet formation, homologous chromosomes are brought into close physical proximity, significantly enhancing their search efficiency and facilitating faster pairing and synapsis [101,102]. Additionally, recombination proteins, such as SPO11, RAD51, and DMC1, play crucial roles in single-strand invasion involved in pairing and synapsis [103,104]. Once homologous chromosomes have achieved a stable structure, known as the synaptonemal complex (SC), the cell formally enters the pachytene stage. During this phase, a characteristic checkpoint eliminates defective spermatocytes that exhibit an incorrect number of double-strand breaks (DSBs), erroneous synapsis, or failed meiotic sex chromosome inactivation (MSCI) (Figure 2) [105,106,107].

Simultaneously, during the single-strand invasion mediated by recombination proteins, single-stranded DNA (ssDNA) pairs with its homologous sequence, forming specialized structures called displacement loops (D-loops). These D-loops primarily resolve into non-crossovers (NCOs), with only a small subset resulting in reciprocal exchanges of flanking chromosome arms, referred to as crossovers (COs). Crossover events, also known as chiasmata, are essential for sexual reproduction, as they induce genetic diversity through chromosomal exchange and ensure the accurate separation of homologous chromosomes by providing a physical connection between maternal and paternal homologs [108,109,110,111]. Following meiotic recombination, the synapsis complex begins to disassemble during the diplotene stage. By metaphase, the entire bivalent—homologous chromosomes linked by chiasmata—aligns along the metaphase plate, ready to be randomly segregated to opposite poles [103,112].

Depletion of SRSF1 in pro-spermatogonia prevents the establishment of a spermatogonial stem cell (SSC) pool. Another study invalidated SRSF1 in undifferentiated and differentiating spermatogonia and revealed its novel role in meiosis, where deletion of SRSF1 resulted in arrest at the pachytene stage. Lei et al. confirmed that SRSF1 directly regulates the alternative splicing of *Stra8* and affects *Dazl*, *Dmc1*, *Mre11a*, *Syce2*, and *Rif1* indirectly, all of which are critical for meiosis [36].

Breast-cancer-amplified sequence 2 (BCAS2) is essential for pre-mRNA splicing in spermatogonia and regulates spermatogenesis-related genes, specifically *Dazl*, *Ehmt2*, *and Hmga1*. After the ablation of BCAS2 in pro-spermatogonia, the spermatogonia appear grossly normal, but very few meiocytes can be observed, indicating an arrest in meiosis initiation [37]. The phenotype resulting from BCAS2 knockout in pro-spermatogonia is similar to that observed with Dazl knockout. BCAS2 knockout leads to a distinct shift from full-length *Dazl* to exon-8-deleted *Dazl* with exon 7 retention, resulting in the significant decrease in DAZL protein level. DAZL, a member of DAZ (deleted in azoospermia) family, typically targets the 3′UTR of specific transcripts, including *Sycp3*, *Mvh*, *Tpx-1*, and *Tex19.1*, thereby regulating the initiation of their translation [38,39,40].

SAM68, a ubiquitous protein with stage-specific expression during meiosis and haploid phase, participates in multiple pathways to regulate alternative splicing. The loss of SAM68 leads to reduced spermatocytes of late prophase and continuous apoptotic haploids [32]. Firstly, SAM68 interacts with phosphorylated RNA polymerase II and other splicing factors to form a complex, which is essential for the splicing of *Sgce* exon 8 [113]. Secondly, SAM68 collaborates with U1snRNP to prevent premature transcript termination by promoting its recruitment to the alternative last exon of target genes [114]. HnRNPH, a hnRNP family member, always suppresses splicing events by competing with other splicing factors, including SAM68. There is an interesting example illustrating how these antagonistic factors coordinate alternative splicing (AS) events in the complex network of transcripts. It is well established that double-strand breaks (DSBs) are catalyzed by SPO11, a topoisomerase-like protein [115]. There are two isoforms of SPO11—SPO11β and SPO11α—determined by the inclusion or skipping of exon 2 [116]. DSBs on autosomes are primarily induced by SPO11β, while those on sex chromosomes depend on SPO11α. The expression of these two isoforms is regulated by the modulation of RNA polymerase II and the recruitment of splicing factors such as SAM68 and hnRNPH [33,35]. In early meiosis, when whole-genome-wide DSBs occur and progressively decrease, the rapid elongation rate of RNA polymerase II promotes the recruitment of SAM68. This, in turn, facilitates the inclusion of exon 2 in pre-mRNA, resulting in the production of SPO11β. In late prophase, as DSBs on autosomes are repaired and only persist on sex chromosomes, the slower elongation speed of RNA polymerase II enhances the recruitment of hnRNPH, competing with SAM68 and advocating exon 2 skipping pre-mRNA and SPO11α splicing. Furthermore, mice that express only SPO11β exhibit deficiencies in sex chromosome pairing, which heightens the risk of aneuploidy—a major contributor to human disorders such as Klinefelter syndrome [117,118]. Disruption of SPO11α leads to defective homologous synapsis in male mice and results in significant spermatocyte death [35].

Later, Feng et al. confirmed that upon deletion of hnRNPH1 (also known as hnRNPH), the exon 2 of SPO11 remained retained in late meiosis, resulting in the related phenotype of sex chromosome asynapsis [33]. Meanwhile, beyond regulating AS of Spo11 in spermatocytes, the group also revealed other critical roles of hnRNPH1 during spermatogenesis. In Sertoli cells, hnRNPH1 interacts with splicing factors PTBP1 to manage the AS of pre-mRNA for target genes functionally associated with cell adhesion. Moreover, it collaborates with the androgen receptor to mediate the transcription levels of various genes linked to cell–cell junctions and the EGFR pathway by binding directly to their promoters in Sertoli cells. In germ cells, hnRNPH1 recruits PTBP2 and SRSF3 to facilitate AS of genes, including *Spo11*, *Cpeb1*, *Tcf7l2*, and *Rbm5*. Notably, many abnormal AS events triggered by the ablation of hnRNPH1 resemble those observed in PTBP2 knockout models. Consequently, the phenotypes of hnRNPH1-null testes are similar to those in PTBP2-null male mice, characterized by an increased number of apoptotic spermatogenic cells and premature release of these cells into the lumen, along with disrupted F-actin distribution. This indicates impaired cell adhesion and compromised germ–Sertoli-cell interactions, which can be attributed to Sertoli cell polarity [33,46].

RBMXL12, also known as hnRNPGT, is expressed exactly during and immediately after meiosis. In Rbmxl2-null testes, there are almost no post-meiotic cells, indicating a complete meiotic block with only rare instances of completed meiosis, rather than a gradual decline in round spermatids. RBMXL2 controls splicing patterns during meiosis, particularly ensuring the accuracy of splice site selection. Specifically, it inhibits the selection of abnormal splice sites and prevents the inclusion of hidden and premature terminal exons [41].

DAZAP1, also belonging to the hnRNP family, plays a crucial role in regulating the splicing of the transcripts such as *Crem*, *Crisp2*, and *Pot1a*, thereby impacting spermatogenic function. Abnormal splicing can lead to the loss of *Pot1a*, which affects telomere integrity and partially explains the growth retardation seen in DAZAP1-deficient mice. The absence of DAZAP1 in the testis results in spermatogenesis halting just prior to meiosis, leading to a lack of haploid spermatids [42].

## 5. Alternative Splicing in Maturation of Haploid Spermatids

After sophisticated meiosis, spermatogenesis comprises spermiogenesis and spermiation, which, respectively, refer to development of post-meiotic male gamete and the release of sperm [119]. During these final stages, haploid cells undergo significant programmed transformations, including chromatin remodeling, elongation of round spermatids, flagella and acrosome formation, and cytoplasmic elimination [119,120]. In the first step of nuclear condensation, the nucleosomal histones in spermatids are gradually replaced by transition nuclear proteins, which are then substituted by protamines, the principal nucleosomal proteins in spermatozoa. This transformation allows for the genetic material to be tightly and orderly compacted, resulting in smaller and more streamlined spermatozoa [121,122].

Before round spermatids differentiate into elongating and elongated spermatids, there are eight distinct sub-phases characterized by changes in acrosome morphology. During the elongation process, organelles undergo reorganization, the sperm tail develops, and mitochondria align along the midpiece of the flagellum to form the sheath. Each of these steps is crucial for the maturation of haploid gametes and male fertility, requiring well-organized gene expression. However, transcription activity ceases around mid-spermiogenesis, with translational control taking precedence in the later stages of spermiogenesis [123,124,125]. As nuclear condensation limits transcription, genes related to spermiogenesis are synthesized in advance and stored as messenger ribonucleoproteins (mRNPs) with suppressed translational activity, ready for activation when needed. Despite significant advancements in our understanding, there remains confusion regarding whether these processes are coordinated at the transcriptome-wide level and how repressed mRNAs are activated for translation [120,122]. Here, combining existing studies, this manuscript offers a tentative glimpse into the landscape of how AS play a role in post-meiosis phase (Figure 2).

MRG15, a MORF-related gene on chromosome 15 that binds to chromatin and regulates pre-mRNA splicing of *Tnp2* by recognizing the methylation of H3K36, which promotes the recruitment of splicing factors during spermatogenesis. Specifically, MRG15 facilitates epigenetic modifications that recruit the splicing factors PTBP1 and PTBP2, thereby enhancing alternative splicing. In MRG15-null germ cells, spermatogenesis arrests at the round spermatid stage, despite normal meiosis and histone acetylation [43]. RBM5 serves as a critical splicing regulator in round spermatids, with its RRM2 domain being essential for accurate splicing of target pre-mRNAs during spermiogenesis. RBM5 also interacts with other splicing factors, including SFPQ and hnRNP A2/B1, underscoring its role in the complex splicing network essential for successful spermiogenesis. A missense mutation (R263P) in the RRM2 domain results in a blockage of spermatid differentiation, leading to a significant loss of germ cells and ultimately causing azoospermia [44]. Additionally, the splicing factor U2AF35-like ZRSR1 is involved in recognizing the 3′ splice sites of both U2 and U12 introns. Mutations in ZRSR1 can cause defects in spermatogenesis by altering U12 intron splicing. ZRSR1 may function within both spliceosomes, leading to combined splicing defects, particularly evident in adjacent U2/U12 intron pairs. Deletion of ZRSR1 results in sperm abnormalities, including defects in the neck, midpiece, head, and tail [45].

ACRBP, known as a proacrosin-binding protein, is produced in two forms in mice due to alternative splicing: ACRBP-W and ACRBP-V5. ACRBP-V5 is involved in the formation and configuration of the acrosomal granule during early spermiogenesis, while ACRBP-W maintains the inactive status of proacrosin in the acrosome until acrosomal exocytosis occurs. ACRBP-null spermatids show malformation of acrosome, a defect that can be rescued by the transgenic expression of ACRBP-V5 rather than ACRBP-W [126]. CUG-BP1/CELF1 is a versatile RNA-binding protein that may influence the alternative splicing of unidentified pre-mRNAs in spermatogonia, spermatocytes, or early spermatids, resulting in the production of isoforms necessary for stage 7 of spermiogenesis, which in turn aids in the elongation of round spermatids. Consequently, knockout of CUGBP1 results in arrest at step 7 of round spermatid development and leads to significant apoptosis [127].

PTBP2 is required for the accurate regulation of alternative splicing, contributing to RNA expression modulation during the initial phase of spermatogenesis. Notably, PTBP2-null tubules exhibit round spermatids within the lumen, along with spermatid arrest and the formation of multinucleate cells during initial differentiation [46]. FBXO24 or F-Box Protein 24, associates with splicing factors (SRSF2, SRSF3, and SRSF9) to regulate alternative splicing in round spermatids. Additionally, it interacts with MIWI and SCF subunits to promote MIWI degradation via K48-linked polyubiquitination. FBXO24 in mice results in irregular histone retention, incomplete axoneme structures, enlarged chromatoid bodies, and atypical mitochondrial coiling along the sperm flagella, ultimately leading to male infertility [47]. The human homologous gene TCP11 produces three alternative splicing products known as TCP11a, TCP11b, and TCP11c. TCP11a binds to Outer Dense Fiber 1 (ODF1), a key component of the sperm tail’s outer dense fibers, which are crucial for the unique morphology and functionality of the sperm tail [128].

The absence of TENR leads to defects in sperm head morphology, primarily characterized by blunt acrosomes and tails that are wrapped around the sperm head. These morphological defects contribute to a significant decrease in sperm motility, which in turn affects the sperm’s ability to penetrate the zona pellucida during fertilization [129]. The C-terminus of TENR bears a striking resemblance to the catalytic domain found in adenosine deaminases that act on RNA (ADARs). ADARs carry out RNA editing by transforming adenosine into inosine within double-stranded RNA, further impacting alternative splicing and translation. Considering this structural characteristic, it is speculated that the disruption of TENR may lead to dramatic changes in the AS events involving sperm morphology [129,130]. RANBP9 is highly expressed in the nuclei of spermatocytes and spermatids, where it interacts with key splicing factors like SF3B3 and HNRNPM, as well as poly(A) binding proteins such as PABPC1 and PABPC2 [131,132]. These interactions facilitate the formation of protein complexes that associate with more than 2300 mRNAs, ensuring their proper splicing and expression. The knockout of RANBP9 leads to reduced sperm counts, total motility, and progressive motility, along with various morphological abnormalities in the sperm, including bent, round, or curved heads, and tails that may be coiled or headless. Mechanistically, the mRNA levels of *Tnp1*, *Tnp2*, *Prm1*, and *Prm2* decreased significantly owing to the absence of interaction between RANBP9 and those key splicing factors. Moreover, four male germ-cell-specific mRNAs (*Ddx25*, *Catsper1*, *Catsper4*, and *Klhl10*) were also downregulated [131].

## 6. Conclusions Remarks

Alternative splicing allows certain genes to generate multiple transcripts, leading to protein isoforms with distinct functions. This process underpins the intricate and well-orchestrated molecular and biological mechanisms in organisms. Spermatogenesis relies on coordinated interactions among various molecules, whose expression is stage-specific and enriched in the testis, reflecting precise temporal and spatial regulation. Our manuscript summarizes the latest studies on genes participating in spermatogenesis via alternative splicing modulation (Table 1). We explore the complex mechanisms by which these splicing factors influence gene expression at both the transcript and translation levels. Additionally, we elucidate the specific mechanisms of alternative splicing, including the spliceosome-catalyzed splicing process, the roles of splicing factors in enhancing or inhibiting splicing at certain regions, and the impact of nonsense-mediated decay (NMD) and post-transcriptional modifications such as m6A, which may affect or be affected by alternative splicing during spermatogenesis. Based on current knowledge regarding germ cell development, we categorize genes related to alternative splicing into three groups: those functioning in spermatogonia, spermatocytes, and spermatids (Figure 2). For each category, we detail the biological and molecular roles of individual genes, their interacting target genes and proteins, and the consequences of their depletion.

If any step in the extensive and complicated network goes wrong, it may lead to non-obstructive azoospermia (NOA)—the most severe male infertility. Currently, there are limited treatments for NOA patients [133]. ICSI is an effective and the most popular strategy for patients who have mature spermatozoa [134]. However, not every NOA patient is amenable to ICSI. For those with testis maturation arrest, stem cell therapy offers a promising future, while a lot of advancement is required in clinically practical applications [135]. The first case of autologous grafting of NOA patient testis tissue has been reported with poor outcome, which also reminds people of this unstable treatment’s high expense financially and timely [136]. Exploring novel and effective therapies is extremely urgent for infertile patients. Moreover, clarifying the AS mechanism in spermatogenesis provides a novel insight into gamete development and potential targets for exploring small molecules and antisense oligonucleotides to correct toxic AS events, caused by common mutations in mammalian genetic defects [137,138,139,140]. In one word, identifying more factors to illuminate the intricate AS landscape holds more promise for the future of infertility patients.

However, there are still some limitations to our review. While we mainly focus on the network of AS in germ cells, it is important to recognize that somatic cells also play a crucial role in the finely tuned mechanisms of spermatogenesis. The interaction between Sertoli cells and germ cells exerts a fundamental influence on cell adhesion [34]. Furthermore, communication among Sertoli cells and between Sertoli and germ cells is essential for forming the blood–testis barrier, which is vital for germ cell development [141]. The Leydig cells produce testosterone, a hormone critical for male fertility; its depletion can lead to one of the most male infertility causes—symptomatic hypogonadism [142,143,144]. In a word, it is necessary to explore AS’s functions in each type of somatic cells and their crosstalk. In summary, our manuscript highlights the latest advancements in understanding alternative splicing in spermatogenesis and provides novel insights into male infertility.

## Figures and Tables

**Table 1:** Gene mutations and its specific roles in spermatogenesis dependent on AS.

Gene. Name	Animal Species	Biological Functions	Biological Consequences	Biological Effects	Reference
*UPF2*	*Mice*	Clear mRNAs with premature termination codons (PTCs) and long 3′UTRs Necessary for Sertoli cell development and spermatogenesis in male mice	Ablation of UPF2 in Sertoli cells leads to Leydig-cell-only phenotype	Nine core splicing factors (*Hnrnpa1*, *Hnrnpc*, *Hnrnpd*, *Hnrnph1*, *Hnrnpk*, *Hnrnpl*, *Ptbp2*, *Srsf11*, and *Srsf9*) are dysregulated.	[15]
*TDRD6*	*Mice/Human*	Required for a proper chromatoid body (CB) architecture Essential for UPF1 localization to CBs, for UPF1-UPF2 and UPF1-MVH interactions	Damaged spermiogenesis characterized by aberrant localisation of CB components and impaired acrosome biogenesis	UPF1 binding to mRNAs carrying long 3′ UTR is disrupted upon TDRD6 deletion—thereby dysfunction of the CB and mis-localization of UPF1—and contributes to increased mRNA stability via decreased degradation.	[16,17]
*METTL3*	*Mice*	METTL3 forms a stable heterodimer with METTL14, facilitating m6A deposition on specific RNAs in the nucleus.	Lack of spermatogonial stem cel l(SSCs)	Decreased exon inclusion level of aberrant AS events of several key genes (*Dazl*, *Sohlh1*, *Cdk11b* and *Nasp*)	[18]
*METTL16*	*Mice*	METTL16 installs the N6-methylation to A43 of U6 snRNA	Impaired spermatogonia differentiation and compromised meiosis initiation	Increased skipping of exons19-21 in *Stag3* mRNA and increased retention of intron 2 in *Ddb2* mRNA	[19]
*ALKBH5*	*Mice*	ALKBH5 is necessary to generate longer 3′UTR mRNAs with correct m6A modification in the post-meiosis phase.	Depletion of pachytene spermatocytes and round spermatids	Exons skipping/inclusion (ESI) appeared to be up-regulated, and intron skipping/retention (ISR) was drastically down-regulated.	[20]
*SRSF10*	*Mice*	Regulate accurate alternative splicing Vital for maintaining alternative-splicing (AS) homeostasis during spermatogenesis	Defected differentiation of progenitor spermatogonia; impaired meiosis initiation	Genes involved in DSEs play crucial roles in RNA metabolism, meiosis, basic biological process, and translation regulation, such as *Dazl* (increased exon inclusion), *Sycp1* (increased exon skipping), *Kit* (A5SS), *Exo1* (A5SS), *Ret* (alternative last exon), and *Cdc7* (increased exon inclusion).	[21]
*SRSF1* (Deletion in prospermatogonia)	*Mice*	SRSF1 is crucial for the homing of precursor spermatogonial stem cells (SSCs) and the survival of spermatogonia by directly binding and regulating the expression levels of TIAR through an alternative splicing pathway.	Sertoli-cell-only syndrome	The expression of spermatogonia-related genes (e.g., *Gfra1*, *Pou5f1*, *Plzf*, *Dnd1*, *Stra8*, and *Taf4b*) was significantly reduced.	[22]
*SRSF2*	*Mice*	SRSF2 regulatory networks play critical roles in several major events including reproductive development, spermatogenesis, meiotic cell cycle, synapse organization, DNA recombination, chromosome segregation, and male sex differentiation	Arrested spermatogonia differentiation	The exon skipping of key genes like *Stag3*, *Stra8*, and *Atr* are increased, and their mRNA abundance levels are decreased.	[23]
*BUD31*	*Mice*	Component of complex B of mammalian spliceosome, required for spliceosome assembly and catalytic activity	Impaired spermatogonial stem cell (SSC) self-renewal and meiosis arrest	The retention of the first intron of *Cdk2* pre-mRNA and decreased expression of *Cdk2* at post-transcriptional level	[24,25]
*CWF19L2*	*Mice*	Functioning particularly and necessarily in the final stage of the spliceosome cycle to form a complex with intron lariat Modulate the alternative splicing of other splicing factors, like RBFOX1, leading to widespread changes in alternative splicing levels	Essential for the initiation of meiosis but little effect on spermatogonia	Disrupted regulation of alternative splicing by directly binding to genes involved in spermatogenesis (such as *Znh1t*, *Btrc*, and *Fbxw7*) and RNA splicing (including *Rbfox1*, *Celf1*, *and Rbm10*)	[26]
*UHRF1*	*Mice*	UHRF1 directly binds to U1, U2, and U4 snRNPs and interacts with a wide array of ribonucleoproteins (RNPs) to modulate the alternative splicing of genes related to spermatogonial development.	Sertoli-cell-only syndrome	Exon-skipped *Tle3* mRNA may be decayed, and the protein expression level of *TLE3* was decreased.	[27]
*HnRNPU* *(absence in Sertoli cells)*	*Mice*	HnRNPU influences pre-pubertal Sertoli cell proliferation, development, postnatal maturation, and the migration and differentiation of prospermatogonia	Testis atrophy and degradation of seminiferous tubules	Decreased recruitment binding to Sox8 and Sox9’s promoter region, diminishing their expression Interaction between hnRNPU and SOX9 and WT1 disappeared, which is necessary for Sertoli cells evolution	[28]
*HnRNPU* (loss in prospermatogonia)	*Mice*	Ditto	Arrest at the transition from T1-ProSG to T2-ProSG; disrupted migration of prospermatognia.	Vrk1, Slx4, and Dazl (related to cell cycle) suffered abnormal alternative splicing. Interact with RNA-binding protein and bind to precursor mRNA to regulate its alternative splicing during spermatogonial development	[29]
*PTBP1*	*Mice*	PTBP1 regulates cellular development by tuning post-transcriptional mRNA modification such as alternative splicing (AS) or mRNA stabilization.	20% of the tubules showing disorganization	More than 200 differentially expressed genes, as well as 85 genes with altered AS, including *Nano3*, due to the loss of PTBP1	[30,31]
*NANO3*	*Mice*	NANO3 plays an important role in the maintenance and survival of primordial germ cells (PGCs).	Impaired pro-spermatogonia differentiation	Both Bax-dependent and -independent apoptotic pathways are activated.	[31]
*SAM68*	*Mice*	SAM68 is able to integrate intracellular signals and RNA processing.	Reduced spermatocytes of late prophase and continuous apoptotic haploids	The highly significant changes in expression of genes involved in cell cycle and cell death pathways might account for the defects in meiotic divisions and the increased apoptosis observed in *Sam68*−/− testes.	[32]
*hnRNPH1*	*Mice*	HnRNPH1 interacts with splicing factors PTBP1 (in Sertoli cell) and SRSF3 and PTBP2 (in germ cell) to manage the alternative splicing (AS) of pre-mRNA for target genes functionally associated with cell adhesion.	Defective synapsis and cell–cell junction	PTBP2 and SRSF3 protein significantly decreased in hnRNPH1 cKO germ cells compared with that in the control, thereby causing significant disrupted transcriptome and proteome related to spermatogenesis.	[33,34]
*SPO11*	*Mice*	SPO11 catalyzes double-strand breaks (DSBs) and participate in recombination.	Disruption of SPO11α leads to defective homologous synapsis in male mice and results in significant spermatocyte death	Deffective DSBs formation on sex chromosomes	[35]
*SRSF1* (loss in advanced germ cells)	*Mice*	SRSF1 plays a key role in the establishment of a spermatogonial stem cell (SSC) pool and meiosis.	Arrested at the pachytene stage	Dysfunctional alternative splicing of *Stra8* and affects *Dazl*, *Dmc1*, *Mre11a*, *Syce2*, and *Rif1* indirectly, all of which are critical for meiosis	[36]
*BCAS2*	*Mice*	BCAS2 is a core component of the CDC5L/Prp19 complex, which is highly conserved and involved in the assembly and conformation of the spliceosome, especially important for the catalytic activation of the spliceosome.	Arrested in meiosis initiation	The aberrant splicing patterns in *Dazl*, *Ehmt2*, and *Hmga1*, which are functional genes in mouse spermatogenesis	[37]
*DAZL*	*Mice/Human*	DAZL plays essential roles for development and sexual differentiation of primordial germ cells (PGCs).	Arrested in meiosis initiation	Typically target the 3′ UTR of specific transcripts, including *Sycp3*, *Mvh*, *Tpx-1*, and *Tex19.1*, thereby regulating the initiation of their translation	[38,39,40]
*RBMXL12*	*Mice*	Bind to RNA molecules and only express during and after meiosis in humans and most other mammals. Bind to other proteins that are known to be involved in controlling splicing of RNAs.	Almost no post-meiotic cells	Control splicing patterns during meiosis, particularly ensuring the accuracy of splice site selection. Specifically, it inhibits the selection of abnormal splice sites and prevents the inclusion of hidden and premature terminal exons	[41]
*DAZAP1*	*Mice*	Belongs to hnRNP family and impacts spermatogenic function.	Growth retardation, and spermatogenesis halting just prior to meiosis	Play a crucial role in regulating the splicing of the transcripts such as *Crem*, *Crisp2*, and *Pot1a*	[42]
*MRG15*	*Mice*	Regulate pre-mRNA splicing of *Tnp2* by recognizing the methylation of methylated histone H3 lysine 36 (H3K36), which promotes the recruitment of splicing factors during spermatogenesis.	Arrested at the round spermatid stage	Recruit the splicing factors PTBP1 and PTBP2, thereby enhancing alternative splicing.	[43]
*RBM5*	*Mice*	Its RRM2 domain being essential for accurate splicing of target pre-mRNAs during spermiogenesis	A missense mutation (R263P) in the RRM2 domain results in ultimate azoospermia	Interact with other splicing factors, including SFPQ and hnRNP A2/B1	[44]
*ZRSR1*	*Mice*	*Zrsr1* encodes a protein that is highly homologous to splicing factor U2AF, which is composed of U2AF65 and U2AF35, playing critical roles in recognizing the polypyrimidine tract and the 3′ splice site AG, respectively.	Sperm abnormalities	Increased intron inclusion of both U2- and U12-type introns, including genes with vital functions in spermatogenesis	[45]
*PTBP2*	*Mice*	Required for the accurate regulation of alternative splicing, contributing to RNA expression modulation during the initial phase of spermatogenesis	Spermatid arrest	PTBP2 deletion disrupts the expression of AS isoforms from a functional network of related genes with roles in membrane remodeling, protein transporting, cytoskeletal reorganization, and GTPase-mediated signaling, mediating cell polarity, adhesion, migration, and cell–cell communication.	[46]
*FBXO24*	*Mice*	Interacts with MIWI and SCF (SKP1-Cullin-F-box) subunits to promote MIWI degradation via K48-linked polyubiquitination.	Sperm abnormalities	Associate with splicing factors(SRSF2, SRSF3, and SRSF9) to regulate alternative splicing in round spermatids.	[47]

Abbreviation: ESI, exon skipping/inclusion; PTCs, premature termination codons; UTRs, untranslated regions; CB, chromatoid body; SSCs, spermatogonial stem cells; ISR, intron skipping/retention; AS, alternative splicing; RNPs, ribonucleoproteins; PGCs, primordial germ cells; H3K36, methylated histone H3 lysine 36; A5SS, alternative 5′ splice sites; A3SS, alternative 3′ splice sites; SCF, SKP1-Cullin-F-box.
